# Efficacy and safety of intravenous paracetamol in comparison to ibuprofen for the treatment of patent ductus arteriosus in preterm infants: study protocol for a randomized control trial

**DOI:** 10.1186/s13063-016-1294-4

**Published:** 2016-04-02

**Authors:** Carlo Dani, Chiara Poggi, Fabio Mosca, Federico Schena, Gianluca Lista, Luca Ramenghi, Costantino Romagnoli, Enrica Salvatori, Maria Teresa Rosignoli, Paola Lipone, Alessandro Comandini

**Affiliations:** Department of Neuroscience, Psychology, Drug Research and Child Health, Careggi University Hospital of Florence, Largo Brambilla 3, 50134 Firenze, Italy; Division of Neonatology, Careggi University Hospital of Florence, Largo Brambilla 3, 50134 Firenze, Italy; Neonatal Intensive Care Unit, Department of Mother and Infant Science, Fondazione IRCCS “Ca’ Granda” Ospedale Maggiore Policlinico, University of Milan, Via Della Commenda 12, 20122 Milano, Italy; Division of Neonatology, “V. Buzzi” Children Hospital of Milan, Via Castelvetro 22, 20154 Milan, Italy; Department of Neonatology Obstetrics and Neuroscience, G. Gaslini Children’s University Hospital of Genova, Via Gerolamo Gaslini 5, 16147 Genova, Italy; Division of Neonatology, Catholic University of Rome, Largo Agostino Gemelli 8, 00168 Roma, Italy; Angelini S.p.A. - Piazzale della Stazione, 00071 S. Palomba -Pomezia, Roma Italy

**Keywords:** Paracetamol, Patent ductus arteriosus, Preterm infant

## Abstract

**Background:**

Patent ductus arteriosus (PDA) is one of most common complications in preterm infants. Although ibuprofen represents the first choice for the closure of PDA, this treatment can cause severe gastrointestinal and adverse renal effects and worsen platelet function. The successful closure of the PDA with paracetamol has been recently reported in several preterm infants, and the safety of paracetamol for this use has been suggested by the available data.

**Methods/design:**

We present the design of a randomized, multicenter, controlled study, whose aim is to assess the effectiveness and safety of intravenous paracetamol in comparison to intravenous ibuprofen for the treatment of PDA in preterm infants. A total of 110 infants born at 25^+0^ to 31^+6^ weeks of gestational age will be enrolled and randomized to receive paracetamol or ibuprofen (55 patients per group) starting at 24–72 h of life. The primary endpoint of the study is the comparison of the PDA closing rate observed after a 3-day course with paracetamol or ibuprofen. The secondary endpoints include the closure rate of PDA after the second course of treatment with ibuprofen, the re-opening rate of the PDA, the incidence of surgical ligation, and the occurrence of adverse effects.

**Discussion:**

The results of this study will provide new information about the possible use of paracetamol in the treatment of PDA. Paracetamol could offer several important therapeutic advantages over current treatment options, and it could become the treatment of choice for the management of PDA, mainly due to its more favorable side effect profile.

**Trial registration:**

Clinicaltrials.gov NCT02422966.

Eudract no. 2013-003883-30.

**Electronic supplementary material:**

The online version of this article (doi:10.1186/s13063-016-1294-4) contains supplementary material, which is available to authorized users.

## Introduction

### Background and rationale

The patency of ductus arteriosus is a frequent complication in preterm infants suffering from respiratory distress syndrome (RDS), and 60 to 70 % of preterm infants at <28 weeks of gestation receive medical or surgical therapy for a PDA [1]. Neonates with a left-to-right shunt through the ductus complicating their RDS have a higher respiratory failure; lower survival rate; and an increased risk of intraventricular hemorrhage (IVH), bronchopulmonary dysplasia (BPD), and necrotizing enterocolitis (NEC) [2]. Therefore, closure of the PDA is indicated before a significant left-to-right shunting occurs.

The current treatment of PDA encompasses two steps: the first is its pharmacological treatment with a nonsteroidal anti-inflammatory drug (NSAID), and the second, in case of medical treatment failure, is surgical ligation, which should be avoided if possible because of the associated severe complications [3]. Standard medical therapy for PDA closure mainly involves either indomethacin or ibuprofen. Both are successful in promoting ductal closure in 70–80 % of cases [2, 4, 5]. However, these drugs can cause severe adverse effects involving the gastrointestinal apparatus, kidney function, and platelet aggregation, thereby inducing the development of gastrointestinal perforations, acute renal failure, and bleeding disorders [6–9]. Therefore, although ibuprofen appears to be the drug of choice at present for PDA pharmacological closure because it shows fewer side effects compared to indomethacin [4], it does not represent the ideal drug because of its sub-optimal safety profile [4, 10, 11] and because of its approximately 30 % failure rate [8–10]. Some concerns have been raised about a possible increased risk of hyperbilirubinemia in ibuprofen-treated infants due to its displacement property of bilirubin from albumin [7], although this effect has not been confirmed [12].

Thus, the pharmacological treatment of PDA remains troublesome [1], and to reduce the occurrence of adverse effects and the need for surgical closure advances in this field should pursue the objective of identifying other drugs suitable for preterm infants that are safer and more effective than ibuprofen.

The successful closure of PDA with paracetamol has been recently reported in several preterm infants, without signs of toxicity [13–22]. A total of 74 preterm neonates with PDA have been treated with paracetamol (oral or intravenous), with closure being obtained in 66 (89 %) neonates without the development of adverse effects (Table 1) [13–22]. Two randomized, single-center, controlled studies compared the effectiveness and safety of oral paracetamol to ibuprofen for the treatment of PDA in preterm infants [23, 24]. Both studies demonstrated that the paracetamol PDA closure rate was similar to that of the ibuprofen (81.2 versus 78.8 % [23] and 72.5 versus 77.5 % [24], respectively). Moreover, Dang et al. [23] found a lower rate of gastrointestinal bleeding and hyperbilirubinemia in the paracetamol than in the ibuprofen group, but these findings were not confirmed by Oncel et al. [24] (Table 2). Interestingly, only two case series [15, 21] have reported the successful effect of intravenous paracetamol for closing PDA in 18 preterm infants. We believe that this point may be very relevant because the intravenous route is certainly more suitable than the enteral route in the preterm infant, whose enteral drug absorption is often uncertain or and who frequently develops feeding intolerance and thus cannot be treated with oral drugs. Therefore, a demonstration that intravenous paracetamol is effective in the closure of PDA could benefit many patients.

**Table 1:** Case series of infants treated with paracetamol for patent ductus arteriosus (PDA)

Author Year	Number of patients	GA (weeks)	BW (g)	Postnatal age (days)	Daily dosage/administration route	No. of PDA closure	Safety issues
Hammerman 2012 [13]	5	26–32	720–1210	3–35	60 mg/kg oral	5/5	No toxicity observed.
Oncel 2013 [14]	8	27–34	630–2970	5–27	60 mg/kg oral	7/8	Pre-treatment and post-treatment liver enzymes were normal in all patients.
Oncel 2013 [15]	10	24–30	590–990	2–15	60 mg/kg intravenous	10/10	No adverse effects were observed. Pre-treatment and post-treatment liver enzymes were normal in all patients.
Yurttutan 2013 [16]	6	26–33	920–1600	3–7	60 mg/kg oral	5/6	No adverse side effects were observed. Pre-treatment and post-treatment liver enzymes and bilirubin were normal in all patients.
Ozdemir 2013 [17]	7	23–32	620–1615	20–47	60 mg/kg oral	5/7	No side effects, no hepatotoxicity, and no abnormalities in the hematologic and biochemical analyses.
Sinha 2013 [18]	10	27–33	800–1380	4–7	45 mg/kg oral	10/10	No side effects related to paracetamol.
Jasani 2013 [19]	6	28–31	1040–1235	2–8	60 mg/kg oral	6/6	No adverse effects. Pre-treatment and post-treatment liver enzymes were normal in all neonates.
Kessel 2014 [20]	7	26–30	789–1322	not available	60 mg/kg oral	7/7	No side effects related to paracetamol.
Terrin 2014 [21]	8	24–28	551–897	38–94 h	60 mg/kg intravenous	6/8	No side effects related to paracetamol.
Nadir 2014 [22]	7	24–27	656–951	2–22	60 mg/kg oral	5/7	No abnormalities in hematologic and biochemical analyses.

**Table 2:** Randomized controlled trials comparing the effectiveness of oral paracetamol to oral ibuprofen in closing patent ductus arteriosus (PDA)

Author Year	Number of patients	GA (weeks)	Mean postnatal age	Treatment dosage	Results
Dang 2013 [23]	160	≤ 34	≤ 14 days	Paracetamol: 15 mg/kg every 6 h for 3 days	Overall, PDA closure occurred in 65 patients (81.2 %) in the paracetamol group and in 63 patients (78.8 %) in the ibuprofen group (*P* = 0.693).
				Ibuprofen: 10 mg/kg followed by 5 mg/kg after 24 and 48 h	The rate of gastrointestinal bleeding and hyperbilirubinemia was significantly higher in the ibuprofen group with respect to the paracetamol group (*P* < 0.05). No significant differences were found for other major adverse events.
Oncel 2014 [24]	90	≤ 30	48–96 h	Paracetamol: 15 mg/kg every 6 h for 3 days	After the first treatment course, the PDA closed in 29 (72.5 %) patients enrolled in the paracetamol group versus 31 (77.5 %) patients assigned to the ibuprofen group (*P* = 0.6). The cumulative closure rates after the second treatment course were high in both groups: only 1 patient (2.5 %) in the paracetamol group and 2 patients (5 %) in the ibuprofen group required surgical ligation.
				Ibuprofen: 10 mg/kg followed by 5 mg/kg after 24 and 48 h	Bilirubin and liver enzyme levels before and after each treatment course were not significantly different between groups. No patient showed oliguria.

Presumably, the beneficial effect of paracetamol in the closure of PDA is mediated through the ability of paracetamol to inhibit in vivo prostaglandin synthesis [25]. Traditional nonsteroidal anti-inflammatory drugs (NSAIDs) compete with the arachidonic acid substrate for the active site of the cyclooxygenase (COX) subunit of Prostaglandin H synthase, block access to the substrate, and, thereby, reduce the levels of prostaglandin production. The potency of the NSAIDs is therefore influenced by the amount of endogenous substrate present. Once the arachidonic acid has gained access to the active site, however, these drugs will have no further inhibitory effect. Paracetamol also inhibits prostaglandin G/H synthase (PGHS) activity, although the precise mechanism of its action remains controversial. It has been proposed that paracetamol does not access the active site of the enzyme, but rather acts on the peroxidase (POX) segment of the enzyme [26]. Thus, whereas COX is dependent on arachidonic acid concentrations, POX can function at low arachidonic acid levels. On the other hand, POX is activated at tenfold lower peroxide concentrations than COX [27, 28]. In principle, these differences would permit POX to operate under conditions where COX is not activated [29]. Under conditions in which the local peroxide concentrations are low, paracetamol-mediated inhibition of PGHS may be facilitated. As a result, the paracetamol might be expected to be maximally effective under conditions of hypoxia, which, in turn, might be associated with low peroxide levels. In other words, paracetamol is, in theory, ideally situated for treatment in the environment that facilitates patency of the ductus arteriosus. Alternatively, paracetamol has been proposed to selectively inhibit a distinct, less well-known, central isoform of cyclooxygenase (COX-3) [30]. This theory has generally been rejected for several reasons [31], and the very existence of a functional human COX-3 has been questioned. In fact, the interactions between paracetamol and cyclooxygenase remain somewhat vague and complex in nature.

### Objectives

The present study has been designed to assess the efficacy and safety of intravenous paracetamol in comparison to intravenous ibuprofen for the treatment of PDA in preterm infants.

### Trial design

This is a randomized, open-label, parallel-group, ibuprofen-controlled, multicenter, prospective study, involving five Neonatal Intensive Care Units (NICU) in Italy. The trial was designed following the SPIRIT 2013 statement (see Additional file 1). The study was planned as a noninferiority trial. A total of 110 patients are expected to be enrolled in the study and to be randomized to either the paracetamol or ibuprofen group (55 patients per group). Informed parental consent will be obtained before enrollment. Patients who meet the inclusion criteria will start the 3-day course of investigational treatment within 24–72 h of age. If PDA closure is achieved, the patients will receive follow-up examinations; if PDA is still open and not hemodynamically significant, patients will be monitored daily for the next 3 days and, then, will receive follow-up examinations; if PDA is still open and hemodynamically significant, patients in both the groups will be treated with a 3-day course of ibuprofen as a rescue medication. In case of further failure of pharmacological treatment, the management of PDA (i.e., further pharmacological and/or surgical treatment) will be performed following the hospital’s local protocol.

**Fig. 1:**
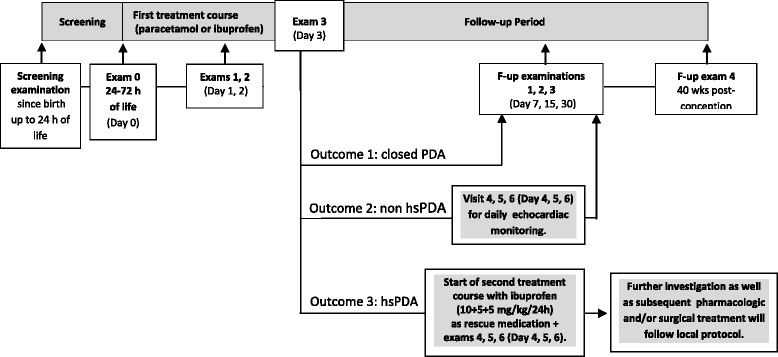
Study procedures flowchart

The full plan of examinations is detailed in the “Interventions” section. The study flowchart is illustrated in Fig. 1.

The duration of the study will be 12 months. The trial will terminate when the last recruited infant performs the last follow-up examination scheduled by the protocol. The study protocol was conceived in October 2013, and approval by the local ethical committees was completed in 2014. However, recruitment could not begin due to the temporary shortage of Pedea® on the market. Recruitment is expected to start by the end of 2015 and to be completed by the beginning of 2017.

## Methods/design

### Study setting

The study will take place at five Italian NICUs in Academic Hospitals. The participating centers are listed below:

- Neonatal intensive Care Unit, Division of Neonatology, Careggi University Hospital of Florence, Largo Brambilla 3, 50134 Firenze, Italy
- Neonatal Intensive Care Unit, Division of Neonatology, Fondazione IRCCS “Ca’ Granda” University Hospital, Via Della Commenda 12, 20122 Milano, Italy
- Neonatal Intensive Care Unit, “V. Buzzi” Children Hospital of Milan, Via Castelvetro 22, 20154 Milan, Italy
- Neonatal Intensive Care Unit, Division of Neonatology, G. Gaslini Children's University Hospital of Genova, Via Gerolamo Gaslini 5, 16147 Genova, Italy
- Neonatal Intensive Care Unit, Division of Neonatology, “A. Gemelli” University Hospital, Largo Agostino Gemelli 8, 00168 Roma, Italy

### Eligibility criteria

#### Inclusion criteria

Inborn infants satisfying the following inclusion criteria will be eligible to participate in the study:

1. Born at 25^+0^ to –31^+6^ weeks of gestation (and)
2. Parental consent has been obtained (and)
3. Echocardiographic evidence of hemodynamically significant PDA between the first 24 and 72 h of life. The diagnosis of hemodynamically significant PDA will be made by echocardiographic demonstration of a ductal left-to-right shunt, with a left atrium-to-aortic root ratio >1.3 or a ductal size >1.5 mm, with those cases in which the closing flow pattern suggests a restrictive PDA being excluded [32, 33].

#### Exclusion criteria

Patients meeting any of the following exclusion criteria will not be eligible to participate in the study:

1. Major congenital malformations and disorders.
2. Fetal hydrops.
3. Life-threatening infection, defined as positive blood culture sample at birth.
4. Echocardiographic evidence of pulmonary hypertension diagnosed when the presence of a right-to-left shunt through the foramen ovale or ductus arteriosus is demonstrated, or the estimated pulmonary pressure from the tricuspid regurgitation jet is more than two-thirds the systemic arterial pressure.
5. Grade 3 or 4 IVH.
6. Serum creatinine concentration >1.5 mg/dl (132 μmol/l).
7. Urine output <1 ml/kg/h during a 24-h collection period or urine output <0.5 ml/kg/h during the first 24 h of life.
8. Platelet count <50,000/mm^3^.
9. Major bleeding, as revealed by hematuria, or blood in the endotracheal aspirate, gastric aspirate, or stools, or consistent blood oozing from puncture sites.
10. Severe liver failure, defined as elevated liver enzymes (ALT/GPT and AST/GOT) >2 times the upper boundary of the normal range. For this kind of population, the following ranges are considered normal: ALT/GPT: 6–50 U/L and AST/GOT: 35–140 U/L [34].
11. Participation in another trial involving any investigational drug.
12. Previous treatment with paracetamol, ibuprofen, or any COX inhibitor, for any purpose.

### Interventions

The study medications will be an intravenous solution (10 mg/ml) of paracetamol (Tachipirina®, Angelini S.p.A., Ancona, Italy) versus an intravenous solution (5 mg/ml) of ibuprofen (Pedea®, Orphan Europe, Puteaux la Defense, France). Patients will be randomly allocated to one of the following treatment groups:

- Group I patients will receive paracetamol intravenous solution, 15 mg/kg per dose (corresponding to 1.5 ml/kg) every 6 h for 3 days, for a total of 12 doses. Paracetamol will be administered in accordance with the clinical practice for the management of pain relief in the newborn [35, 36].
- Group II patients will receive ibuprofen intravenous solution at an initial dose of 10 mg/kg, followed by 5 mg/kg after 24 and 48 h. Pedea® has been selected as the comparator treatment because it represents the only drug authorized in Europe since 2004 for the treatment of a PDA in preterm newborn infants <34 weeks of gestational age. The ibuprofen doses are consistent with those reported in the relevant Summary of Product Characteristics.

Both intravenous drugs will be infused continuously over a period of 15–30 min.

The dose of 15 mg/kg for IV paracetamol was chosen based on previously reported data for paracetamol in the treatment of PDA in preterm newborns, although lower doses are also reported in this population for pain-control purposes [37, 38]. All reported case series of paracetamol for PDA closure was administered orally or intravenously, 15 mg/kg every 6 h for 3 to 7 days [39–44]. No adverse effects related to the drug were reported [39–44], even in patients treated with long courses of paracetamol for 6–7 days [42–44], thus receiving twice as much or more of the total amount of drug than our population. Pharmacokinetics of paracetamol has been poorly investigated so far in preterm newborns. Data from pharmacokinetic studies of paracetamol in the analgesia of preterm newborns were recently included in a population pharmacokinetic analysis [45]. Using a three-compartment structural model for distribution and elimination of the drug, the authors demonstrated that dosage ranges from 11 to 13 mg/kg per dose were appropriate for newborns of 500–2000 g [45]. Moreover, plasma paracetamol concentrations were also measured in one recent study investigating paracetamol for PDA closure [43]. Patients received 15 mg/kg every 6 h for 3 days, and the plasma paracetamol concentration assessed before the fifth and ninth doses and 24 h after treatment termination did not exceed those recommended for analgesia in infants.

Compliance will be defined as full adherence to the protocol. Compliance with the protocol will be ensured by a number of procedures included in the site set-up. The local principal investigator participated in the preparatory meetings in which details of the study protocol and data collection were accurately discussed. All centers received detailed instruction on study procedures and web-based recording data, and contact will be possible for the sponsor study manager or the appointed CRO, which is delegated by the sponsor to perform monitoring activity, to resolve possible difficulties. During the trial, the investigational centers will be periodically contacted by the designated clinical monitor, who will perform on-site visits in order to verify that the trial is conducted in accordance with the protocol, Good Clinical Practice, and all applicable regulatory requirements.

Daily clinical care of the enrolled patients will be performed by the attending physicians in accordance with the common practice at each center. Daily fluid intake will be started at 70–80 ml/kg and gradually increased by 10–20 ml/kg/day on the basis of changes in body weight, serum sodium concentrations, and osmolality, with a target intake of 150–160 ml/kg at the end of the first week of life. In case of hypotension refractory to the fluid-replacement therapy, dopamine and/or dobutamine treatment will be provided. For the treatment of RDS, infants will receive oxygen therapy, respiratory support, and rescue surfactant treatment in order to achieve the following therapeutic targets: PaO_2_ 50–60 mmHg, PaCO_2_ < 65 mmHg, pH > 7.20, and SpO_2_ 90–95 %. Infants will be fed with maternal and/or human milk and/or preterm formula from the first day of life as minimal enteral feeding or for nutritional purposes at the discretion of the physician on duty. For cases in which the infusion of a glucose solution is clinically indicated, the concentration will be chosen in order to maintain an appropriate level of glycemia. In patients with a central venous line or weighing <1500 g (regardless of the type of venous access), amino acids will be administered from the first hour of life, and fat, no later than at 2 days of life. Prophylactic antibiotics will be administered from the time of admission to the NICU and will be stopped after 3–4 days if the results of the bacterial cultures remain negative.

During the study, the use of other NSAIDs will not be allowed for any purposes, and concomitant medications and adverse events will be recorded in the patient’s electronic Case Report Form (eCRF).

### Outcomes

#### Primary outcome measurements

The primary endpoint of the study will be the success rate in closing the PDA using paracetamol in comparison to ibuprofen after the first course of treatment.

#### Secondary outcome measurements

Secondary outcome measurements will include the following:

1. Closure rate of PDA after the first and second day of the first treatment course
2. Closure rate of PDA after the second course of treatment with ibuprofen
3. Re-opening rate of PDA
4. Incidence of surgical ligation
5. Incidence of renal failure, liver failure, and gastrointestinal complications (NEC and isolated perforation) within 30 days. For study purposes, the renal failure is defined as a serum creatinine concentration >1.5 mg/dl (132 μmol/l) and urine output <1 ml/kg/h during a 24-h collection period. Liver failure is defined as elevated liver enzymes more than two times the upper boundary of the normal range (normal ranges: ALT/GPT = 6–50 U/L and AST/GOT = 35–140 U/L) [34].

#### Further collected data

The following data will be recorded for each infant: gestational age (GA); birth weight (BW); birth length; birth cranial circumference; sex; mode of delivery; Apgar score at 5 min; main maternal pathologies; antenatal steroid treatment; vital signs; the highest FiO_2_ and mean airway pressure values; need and duration of noninvasive airway pressure ((nasal continuous airway pressure (NCPAP), biphasic positive airway pressure (BiPAP), nasal intermittent mandatory ventilation (N-IMV), and humidified high flow nasal cannula (HHFNC)) and invasive ventilation respiratory support ((patient-triggered ventilation (PTV), including synchronized intermittent positive pressure ventilation (SIPPV), synchronized intermittent mandatory ventilation (SIMV), pressure support ventilation (PSV), or high frequency oscillatory ventilation (HFOV)); the need for surfactant treatment; daily fluid intake; urine output; concomitant diseases; concomitant treatments; and adverse events.

We will also report the occurrence of sepsis, IVH, periventricular leukomalacia (PVL), BPD, retinopathy of prematurity (ROP), NEC, length of stay in the neonatal intensive care unit (NICU) and hospital, and mortality.

A diagnosis of sepsis will be based on clinical and laboratory data (white cell count, C-reactive protein concentration) and will be confirmed by positive blood cultures [46]. IVH will be graded according to a standard classification system [47]. A diagnosis of PVL will be made when cystic areas are detected by cerebral ultrasonography at 40 weeks post-conception birth [48]. BPD will be defined as the oxygen requirement at 36 weeks post-conception [49]. ROP will be graded according to the international classification of retinopathy of prematurity [50]. NEC will be diagnosed in agreement with classical Bell’s criteria [51]. Patients will be discharged from the NICU to a lower level of care when they no longer need respiratory assistance other than oxygen-therapy and central venous catheters.

### Participant timeline

The patients enrolled in the trial will receive a maximum of 12 clinical examinations: the screening exam from birth up to 24 h of life; the exam 0 at 24–72 h of life (before starting pharmacological treatment); exams 1, 2, 3, 4, 5, and 6 carried out 1, 2, 3, 4, 5, and 6 days after the exam 0; follow-up exams at 7 (±1), 15 (± 2), and 30 (± 2) days after the exam 0; and at 40 weeks of post-conception. A heart ultrasound will be performed at screening and exam 0 to rule out ductal-dependent congenital heart diseases and pulmonary hypertension and to evaluate the PDA. In enrolled patients, echocardiography will be repeated every 24 h during the first and second treatment course and 24 h after the last dose of the treatment. The echocardiography will be repeated at the first three follow-up exams and in case of clinically suspected PDA re-opening. If, at exam 3, the echocardiogram indicates a nonhemodynamically significant PDA, further daily echocardiographic evaluation will be performed for the next 3 days. In cases of PDA, the ductal size and the left-atrium-to-aortic-root ratio will be recorded at each heart ultrasound.

Cardiac ultrasound will be performed by expert personnel, specifically a pediatric cardiologist or a neonatologist who has achieved adequate expertise in newborn heart ultrasound, fulfilling the current recommendations for echocardiography training in the NICU (45). The personnel in charge of cardiac ultrasound will be blind to the randomization arm of the patient for the entire duration of the study period.

Serial cerebral ultrasounds for evidence of possible IVH and/or PVL will be performed at the screening; at the exams 0, 3, and 6; at the follow-up exams 7 (± 1), 15 (± 2), and 30 (± 2) days after exam 0; and at 40 weeks post-conception.

Laboratory tests will be performed at the screening; at exams 3 and 6; and at the follow-up exams 7 (± 1) and 30 (± 2) days after exam 0. Clinical laboratory tests will include the count of red blood cells, white blood cells, and platelets and the serum value measurements of hemoglobin/hematocrit, creatinine, urea nitrogen, total bilirubin, total proteins, liver enzymes, sodium, potassium, and calcium. The laboratory test will be performed on a 1-ml blood sample, obtained by heel, venous, or arterial puncture. Urine output will be monitored during the study by weighing nappies or by urethral catheterization if clinically indicated, beginning with the screening visit.

**Fig. 2:**
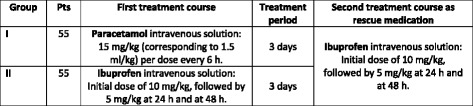
Participant treatment timeline

**Fig. 3:**
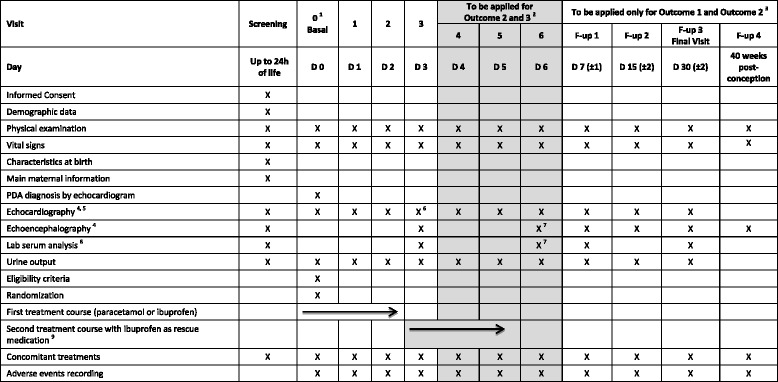
Participant visit timeline. ^1^Visit 0 has to be performed at 24–72 h of age. Visit 0 and the screening visit can be performed in the same day starting at 24 h of life. ^2^ Visit 4, Visit 5, and Visit 6 have to be performed in case the echocardiography highlights a non-hsPDA (Outcome 2) or a persistent hsPDA (Outcome 3) at Visit 3. ^3^ Follow-up visits (F-up 1, F-up 2, F-up 3, and F-up 4) have to be performed only by patients with an ongoing presentation of Outcome 1 and Outcome 2 at Visit 3. ^4^ The following parameters will be recorded: ductal size and the left-atrium-to-aortic-root ratio. ^5^ At Visit 3, in accordance with the echocardiographic evaluation, patients will be differentiated in the following: Outcome 1: patients presenting closed PDA. Outcome 2: patients with non-hsPDA. Outcome 3: patients with persistent hsPDA. ^6^ Procedures applicable only for patients undergoing the second treatment course of ibuprofen as rescue medication. ^7^ Laboratory analyses: hematology (including RBC, WBC, platelets, hemoglobin, and hematocrit), creatinine, blood urea nitrogen, AST/GOT, ALT/GPT, total bilirubin, total protein, sodium, potassium, and calcium

The schedule of the investigational drug administration is summarized in Fig. 2, whereas the visits and exams are summarized in Fig. 3.

### Sample size

The study was conceived as a superiority trial aimed to demonstrate the superiority of paracetamol over ibuprofen on the primary efficacy endpoint of closing PDA after a 3-day course. Assuming a 25 % ibuprofen failure rate in closing PDA [52] and a 5 % paracetamol failure rate (improvement of 20 %), we used the *χ*^2^ test to calculate that a sample size of 49 evaluable patients per group will be necessary to determine a statistically significant decrease of 20 % in the failure rate in the paracetamol group at a two-sided alpha level of 5 % and with a power of 80 %. The sample size was calculated with the NQuery Advisor® software 7.0 and SAS® PROC POWER. Hypothesizing a 10 % drop-out rate of patients who will not complete the first 3-day course of treatment, we planned to enroll 55 infants in each group; thus, 6 more infants were needed for each group, as calculated with N = n/(1−d).

The assumption of a 95 % efficacy of paracetamol was based on previously reported data on the effectiveness of paracetamol in the treatment of hsPDA, ranging from 71 % [17] to 100 % [13, 18–20]. Specifically, the lowest observed efficacy of 71 % was observed in a cohort of newborns who received oral paracetamol after a double-course ibuprofen failure [17], whereas an efficacy of 100 % was reported in four case series [13, 18–20] and of 83–88 % in three case series [14–16]. Overall, despite the high heterogeneity of the reported cases, paracetamol was effective in 93 % (55/59) of the treated infants.

The following populations are defined for statistical analysis: the modified intention-to-treat (m-ITT) population is includes all randomized patients who have completed the first treatment course and have undergone baseline and day 3 echocardiographic assessment; the per protocol (PP) population includes patients from the m-ITT population who have no major protocol violations; and the safety population (SP) includes all patients who take at least one dose of study medication.

### Recruitment

Before patients are entered into the study, the parents (or legal representatives) will be fully informed about the purposes of the study, possible benefits, any potential reasonable risk or discomfort, the expected duration of the newborn’s participation, the clinical and/or functional procedures, as well as the name of the investigator(s) responsible for conducting the trial and his/her direct contact details. Written and oral information will be offered to parents, and sufficient time will be allowed for consent. The newborn will enter the study only after both parents or legal representatives sign the informed consent form. A senior investigator will be available at all times to discuss concerns raised by parents or clinicians during the course of the trial.

A monthly accrual report about the study will be sent to participating centers.

An estimated 680 infants of gestational age 25^+0^ to 31^+6^ weeks will be born during the 12-month period at the participating centers: 160 at the Florence Site, 160 at the Rome site, 160 at the Milan site (Ospedale Maggiore Policlinico of Milan), 100 at the Genoa site, and 100 at the Milan site (“V. Buzzi” Children Hospital of Milan). Of the 270 remaining infants, we assume that 25 % would not be enrolled due to PPHN, major malformations, or the absence of parental consent, thereby resulting in 216 eligible patients. Therefore, no major problems of enrollment completion are expected, as the expected eligible population definitely exceeds the number of patients who must be enrolled.

### Assignment of interventions

#### Sequence generation

Infants at each unit will be randomly assigned in blocks to a treatment group (paracetamol or ibuprofen) in a 1:1 ratio. The allocation sequences consist of computer-generated random numbers.

Since the frequency of the PDA is inversely related to gestational age (GA), the inclusion of patients will be balanced in each treatment group according to the following gestational ages: 25^0^ to 27^+6^ weeks or 28^+0^ to 31^+6^ weeks.

#### Allocation concealment

The enrolled patients will be allocated to the treatment arm using automatically generated, sealed, opaque labels, which will be provided to each participating center by the sponsor to ensure the allocation concealment. Patient enrollment and allocation to treatment will be the responsibility of the neonatologists involved in the study at the different participating centers.

#### Blinding

As the primary endpoint (PDA closure) is objectively detected by echocardiography and cannot be affected by the open label-condition, the study will not be blinded. However, the personnel in charge of the cardiac ultrasound will be blinded to the randomization arm of the patient for the entire duration of the study period. On the other hand, the open-label design is necessary because the different dose schedule of paracetamol and ibuprofen makes the double-blind design inapplicable. However, to minimize bias, strict criteria and definitions for hsPDA will be maintained during the trial.

### Data collection methods

All collected data will be obtained from the clinical records at each clinical examination scheduled by the protocol, as summarized in Fig. 3. No duplicate measurement strategy for laboratory tests will be applied; this will minimize the volume of the blood samples, as the enrolled population will consist of very preterm newborns who would be exposed to a consistent risk of iatrogenic anemia in case of repeated samples. Cardiac ultrasound will be performed by expert personnel, specifically a pediatric cardiologist or a neonatologist who has achieved adequate expertise in newborn heart ultrasound, having fulfilled the current recommendations for echocardiography training in the NICU [25]. Data collection forms are included in the eCRF that was specifically designed for the trial.

The data for patients discontinuing the study will be collected as summarized in Fig. 3.

### Data management

All the collected data will be recorded by the local investigator on a web-based eCRF that was specifically designed for this study by Angelini S.p.A. using SAS PheedIt system. SAS PheedIt is a fully validated web-based application that is compliant with the FDA 21CFR11 and European regulations regarding electronic records and signatures. Data entry screens have been created to capture the clinical data required per protocol. Only authorized site personnel with an assigned personal account and password will have access to the eCRF. Subjects will be identified by sex, birth date, and assigned trial number only, in accordance with personal data protection law.

All study data captured in the study database will be (1) verified against original data records by the clinical monitor during on-site monitoring visits and (2) subjected to quality control and referred back to the relevant center for clarification in the event of inconsistency, missing items, or uncertainty.

The study coordinator and trial statistician will review the generated results for logic, coherence, or problems. Outlier data will be investigated.

### Statistical methods

An analysis of the primary and secondary endpoints of the study will be carried out on the m-ITT and PP populations. The clinical characteristics of the infants in the paracetamol and ibuprofen groups will be described using mean value and standard deviation, median value and range, or frequencies and percentage. Univariate statistical analysis will be performed using the Student *t* test for parametric continuous variables, the Wilcoxon rank-sum test for nonparametric continuous variables, and the χ-square test for categorical variables. A *P* < 0.05 will be considered statistically significant. The type of treatment, clinical characteristics that are most likely associated with the occurrence of PDA refractory to pharmacological closure (i.e., gestational age, birth weight, antenatal steroids, mechanical ventilation, etc.), and variables that will be significantly different between groups (*P* < 0.100) will be included in the multiple logistic regression analysis to assess their independent roles in predicting the closure of PDA. Effect estimates will be expressed as the relative risk (RR) with profile likelihood-based 95 % confidence limits.

Safety and tolerability assessments will be carried out on the SP population.

### Missing data

In order to prevent a large amount of missing data, the follow-up period for the primary outcome of ductal closure was established in the short term for 30 days, whereas the follow-up period was conceived to finish at 40 weeks of gestation, when the enrolled patients are likely to be still admitted to the hospital. The personnel involved in the study will be selected based on their expertise in a previous clinical trial, and all the investigators and study staff will be trained on the importance of the completion of the study period of enrolled patients. Parents will also be informed about this crucial aspect to reduce dropout, and a senior investigator will be available at each site anytime the parents may need further information or clarification during the study period.

### Data monitoring

Data will be monitored by personnel from the “Mediolanum Cardio Research”, Via Carducci 19, 20123 Milan, a society offering support for clinical trials, which is independent from the sponsor.

No interim analyses are scheduled per protocol. The decision to terminate the trial is the responsibility of the sponsor and of the principal investigators of the coordinating center.

### Harms

Safety and tolerability will be assessed by monitoring the frequency and severity of adverse events. Changes from baseline in the physical examination, vital signs, and urine output will be also assessed. Laboratory analyses will be evaluated on the basis of the normal ranges, the investigator’s judgment, and the mean changes from screening.

All adverse events, both nonserious and serious, whether treatment related or not, or expected or unexpected, will be recorded during the clinical trial, starting from receipt of the signature of informed consent to the last follow-up examination scheduled at 40 weeks post-conception age. All serious adverse events will be transmitted in a timely manner (within 24 h from initiation) by the investigator to the sponsor, who is responsible for the relevant notification to all applicable competent authorities in accordance with pharmacovigilance regulations.

## Ethics and dissemination

### Research ethics approval

The study protocol was conceived in October 2013 in accordance with the European Community Guidelines of Good Clinical Practice for Trials on Medicinal Products and the “Declaration of Helsinki” (World Medical Assembly in 1964 and following revisions). The study was approved by the Italian competent authority AIFA (Agenzia Italiana del Farmaco) and by the local ethical committees of the participating centers: the Pediatric Ethical Committee of Tuscany, Ethical Committee of Milan-B Area, Ethical Committee of Milan-C Area, Ethical Committee of Liguria and Ethical Committee of the Catholic University of the Sacred Heart of Rome.

Protocol amendment during the study period, if any, will be submitted for approval to all the aforementioned ethical committees.

### Consent

Before patients are entered into the study, the parents (or legal representatives) will be fully informed about the purposes of the study, possible benefits, any potential reasonable risk or discomfort, the expected duration of the newborn’s participation, the clinical and/or functional procedures, as well as the name of investigator(s) responsible for conducting the trial and his/her direct contact details. Written and oral information will be offered to the parents, and sufficient time will be allowed for consent. The consent will be obtained by the neonatologists involved in the trial at the different participating centers. A senior investigator or a junior investigator with a senior supervisor will be in charge of obtaining the consent.

### Confidentiality

Only authorized site personnel will be authorized to collect patient data for the study purposes and will have access to the eCRF with an assigned personal account and password. Subjects will be identified by sex, birth date, and assigned trial number, during and after the trial, in accordance with personal data protection law.

### Access to data

The principal investigators of each site and the sponsor will have complete access to the final trial dataset, and no contractual agreement exists to limit such access for the investigators.

### Dissemination policy

The results of the trial are expected to be published in a scientific journal and to be presented in medical seminars and conferences. The final reporting will follow the CONSORT Report guidelines (http://www.consort-statement.org).

## Discussion

Recent results reported on the use of paracetamol in the treatment of PDA are highly promising, but adequately powered, randomized controlled clinical trials are needed to validate these promising observations. The aim of this study is to collect consistent data on the efficacy and safety of intravenous paracetamol, in comparison to ibuprofen, for the treatment of PDA in preterm infants.

The proper timing of the pharmacological treatment of hsPDA in preterm newborns is still a matter of debate, and the clinical practice widely varies among different centers [53]; early treatment may have a beneficial impact on early PDA-related morbility, whereas late delayed treatment prevents overtreatment in those cases of spontaneously closing PDA [54]. Recent data suggest that early ibuprofen treatment of hsPDA at a median age of 3 days does not significantly affect the occurrence of BPD, death, BPD or death, NEC, severe IVH and PVL, in comparison with delayed treatment at median age of 11 days in newborn of 23–32 weeks of gestation, suggesting that late treatment is safe and more favorable in terms of overtreatment reduction [54]. It has been reported that 49 % of all PDA spontaneously close within the first week in newborns with birthweight <1500 g and in 94 % of newborns prior to discharge, suggesting that deferring treatment decisions until at least 1 week of life may avoid unnecessary treatment exposure [55]. In a cohort of newborns <27 weeks of gestational age, treated with an early (0–2 days), intermediate (3–6 days), or late (≥ 7 days) start, the timing of PDA treatment was not associated with a risk of PDA surgery or death [56].

However, only 31 % of infants with a birthweight <1000 g presented spontaneous closure within 1 week [55], and in a cohort of preterm newborns treated with mixed timing of initiation, newborns who experienced failed PDA closure with indomethacin showed significantly delayed treatment initiation in comparison to newborns with successful closure, and newborns treated on day 5 presented a significantly higher failure rate in comparison to those treated on day 3, suggesting that later initiation of treatment may decrease the success rate [57]. Moreover, a randomized controlled trial of very early treatment of PDA within the first 12 h of life in newborns <29 weeks of gestation demonstrated that newborns who received early indomethacin had a lower occurrence of early pulmonary hemorrhage and a trend toward less IVH in comparison to the placebo group, although no differences were detected in the death rate [58]. In fact, very early pharmacological prophylaxis regimens of PDA are still in use, at least in very preterm babies [59].

Paracetamol could offer several important therapeutic advantages over current treatment options, considering that in the neonatal population, it appears well tolerated when used at the analgesic dosing regimen commonly administered in NICU [35]. Finally, paracetamol could become the treatment of choice for the management of hemodynamically significant PDA, mainly due to its more favorable side effect profile. As the participating centers’ practices consists of early treatment of hsPDA during the first 24–72 h of life, any positive results of our study may need to be confirmed in clinics using a late treatment approach before paracetamol may be considered for hsPDA closure independently of the timing of treatment.

### Trial status

Patient enrollment began in December 2015 in one center and in January 2016 in the remaining center.

## Additional file

Additional file 1: The SPIRIT 2013 Statement recommendations for clinical trial protocols. (DOCX 50 kb)

## Abbreviations

BiPAP: biphasic positive airway pressure
BW: birth weight
BPD: bronchopulmonary dysplasia
CMV: continuous mandatory ventilation
eCRF: electronic case report form
GA: gestational age
HFOV: high-frequency oscillatory ventilation
HHFNC: humidified high-flow nasal cannula
IVH: intraventricular hemorrhage
m-ITT: modified intention-to-treat
NCPAP: nasal continuous airway pressure
NEC: necrotizing enterocolitis
NICU: neonatal intensive care units
N-IMV: nasal intermittent mechanical ventilation
NSAID: nonsteroidal anti-inflammatory drug
PDA: patent ductus arteriosus
PP: per protocol
PVL: periventricular leukomalacia
RDS: respiratory distress syndrome
ROP: retinopathy of prematurity
SIPPV: synchronized intermittent positive pressure ventilation
SP: safety population
